# Spatiotemporal evolution characteristics and dynamic efficiency decomposition of carbon emission efficiency in the Yellow River Basin

**DOI:** 10.1371/journal.pone.0264274

**Published:** 2022-03-24

**Authors:** Yuan Zhang, Zhen Yu, Juan Zhang

**Affiliations:** 1 School of Management, China University of Mining & Technology (Beijing), Beijing, China; 2 State Key Laboratory of Precision Measuring Technology and Instrument, Tianjin University, Tianjin, China; 3 Airport College, Binzhou University, Binzhou, China; Universita degli Studi del Molise, ITALY

## Abstract

The Yellow River Basin (YRB) is China’s substantial energy consumption base. The issue of carbon emission efficiency directly affects the ecological protection and high-quality development of the YRB. It is the key to achieving carbon peak in 2030 and carbon neutralization in 2060 (“30.60”) double carbon emission reduction targets. Therefore, taking YRB as the research object, this paper first calculates the carbon emission and the decoupling state in the YRB. Secondly, the super-efficiency slacks-based measurement (SE-SBM) model is combined with the Malmquist index to analyze the temporal and spatial evolution characteristics of YRB’s carbon emission efficiency from static and dynamic perspectives. Thirdly, the dynamic evolution characteristics of carbon emission efficiency are analyzed with the help of the Kernel density function. Finally, the Tobit model analyzes the influencing factors of YRB’s and China’s carbon emission efficiency. The results show that: (1) Among the nine provinces of YRB, the decoupling state between carbon emissions and economic growth in most provinces changes from weak decoupling to strong decoupling, and the decoupling elasticity index shows a fluctuating downward trend. (2) There are significant differences in carbon emission efficiency among provinces, but on the whole, it shows a stable growth trend. The high-value area of carbon emission efficiency is increasing, and the phenomenon of two-level differentiation is improving. The decline of the technological progress index causes the Malmquist index in Qinghai and Ningxia. On the contrary, the rise of the Malmquist index in the other seven provinces is caused by improving the technical efficiency index. (3) Industrial structure, economic development, and industrialization are the main positive factors affecting YRB’s carbon emission efficiency. Urbanization level, green development level, and energy consumption level are the leading negative indicators hindering YRB’s improvement of carbon emission efficiency. Therefore, targeted emission reduction suggestions should be formulated according to YRB’s resource endowment and development stage characteristics.

## 1. Introduction

The United Nations Climate Change conference in 2009 set the goal of human Climate Governance action that the global average temperature is no higher than 2 degrees Celsius before the industrial revolution. The Paris climate change conference in 2015 also proposed to control the rise of the global average temperature within 2 degrees Celsius compared with the level before industrialization and strive to control the temperature rise within 1.5 degrees Celsius. Article 4 of the Paris agreement states that a balance needs to be reached between anthropogenic greenhouse gas emissions by sources and removals by sinks in the second half of the 21st century, zero emissions by 2050 [1, 2]. Given this international carbon emission reduction background, as the largest developing country, China has also successively put forward the relevant goals of achieving the "30.60" double carbon goal.

The Yellow River is the second-longest river in China and the fifth-longest river globally. It traverses the three strategic regions of East, middle, and West China. The YRB is one of the most important in one of the "one belt, one road" initiatives, rich in energy resources and prominent in ecological status. It is also a key area for national poverty alleviation and regional coordinated development [3]. The Yellow River and its coastal basin are among the most important birthplaces of the Chinese nation and the main battlefield of national ecological civilization construction. The YRB occupies a critical strategic position in China’s economic and social development and environmental security barrier structure and has dramatically impacted human civilization [4, 5]. The YRB is rich in mineral resources, and coal, oil, and natural gas occupy a crucial position: China’s vital energy, chemical industry, raw materials, and fundamental industrial base [6]. Therefore, the comprehensive energy efficiency and carbon emission efficiency of the YRB directly affect the ecological protection and high-quality development of the YRB. As an essential development region in China, the carbon emission and economic development level of the YRB account for a considerable proportion. Therefore, the solution of the carbon emission efficiency of the YRB is directly related to the realization of China’s "30.60" double carbon goal and then affects the realization process of the global climate change goal.

**Fig 1:**
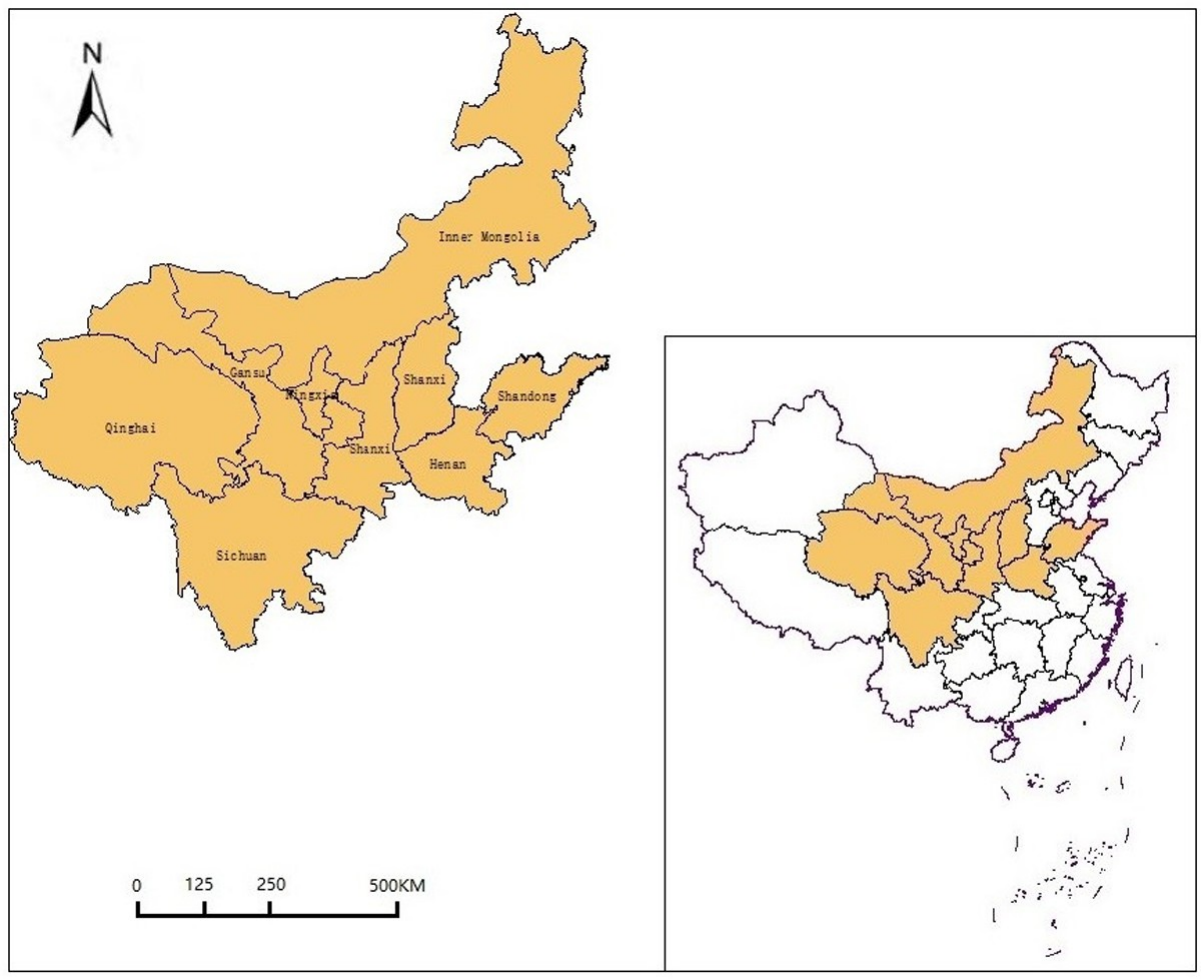
The geographical location of the YRB in China.

**Fig 2:**
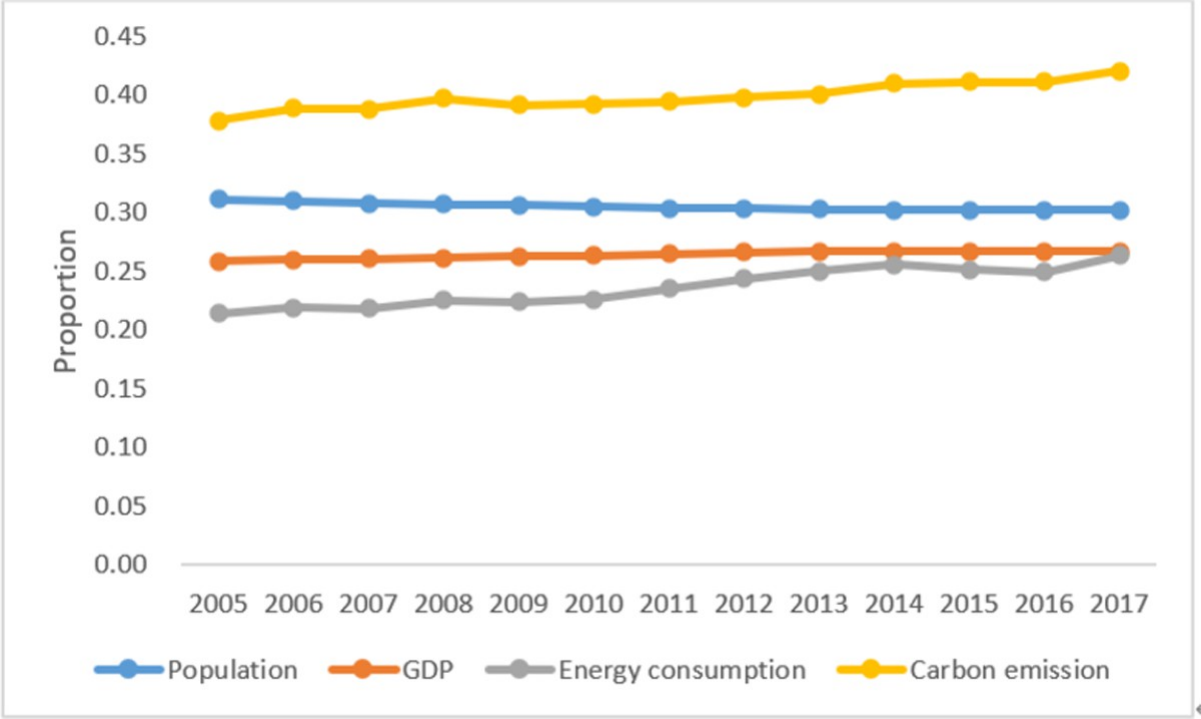
The proportion of the YRB to China. (Data sources: Calculated according to the China Statistical Yearbook from 2006 to 2018).

This paper selects nine provinces in the YRB as the research object to study the temporal and spatial evolution characteristics and difference analysis of influencing factors of carbon emission efficiency in this region. The YRB mainly includes Shanxi, Inner Mongolia, Shandong, Henan, Sichuan, Shaanxi, Gansu, Qinghai, and Ningxia, as shown in Fig 1. During the study period from 2005 to 2017, the proportion of the population in YRB decreased slightly from 31.1% to 30.2%, the proportion of total economic volume increased slightly from 25.9% to 26.7%, the balance of energy consumption increased from 21.4% to 26.3%, and the ratio of carbon emission risen from 37.9% to 42.8%, as shown in Fig 2. It can be seen that the carbon emission contribution of the YRB is much more significant than the economic contribution, and it belongs to a high carbon emission area. Therefore, to achieve the 1.5 degrees Celsius goal of the Paris Agreement and China’s "30.60" double carbon emission reduction goal, as an essential energy consumption base in China, the research on the temporal and spatial evolution characteristics of carbon emission efficiency in the YRB region and the main driving factors affecting the region has become the key.

## 2. Literature review

According to the measurement method, the literature research on carbon emission efficiency is mainly divided into single and total factors. In the study of a single factor, some scholars use carbon emission per unit energy consumption [7], ratio of carbon dioxide to GDP [8], carbon dioxide emission intensity [9], per capita carbon dioxide emission [10] to express carbon emission efficiency. Although a single factor is simple and easy to understand in the calculation, it limits inaccurate expression. The direct single-factor calculation cannot be accurately estimated because carbon emission efficiency is essentially the input-output efficiency of economic activities. Based on the idea of the total factor, economic output, energy consumption, capital stock, and labor force are expected output, and gross domestic product (GDP) and CO2 are expected output and unexpected output, respectively. The carbon emission efficiency measurement results are more accurate. On this basis, Ramanathan uses data envelopment analysis (DEA) to calculate the carbon emission efficiency of various countries [11]. Subsequently, many scholars used DEA and its extended model to estimate the carbon emission efficiency of regions and industries [12–16]. In terms of research methods, the results may deviate because the DEA model only focuses on the expected output in economic activities and ignores the unexpected output [17]. Some scholars use improved models when measuring carbon emission performance, such as directed distance function model (DDF) [18], slack based measure (SBM) [19], super-efficiency SBM model (SE-SBM) [20], SBM-DDF model [21], etc.

The SE-SBM model considers expected and unexpected output, so it is widely used in efficiency measurement in various fields. Huang et al. [22] calculated the utilization efficiency of agricultural water resources through the super efficiency SBM model and provided reasonable suggestions for utilizing regional water resources. Long et al. [23] proposed an SBM-DEA model with unexpected output efficiency to measure urban ecological sustainable development. Wang et al. [24] evaluated the efficiency of public cultural services and found the main factors affecting efficiency. Tian et al. [25] assessed the efficiency of sustainable traffic development and put forward schemes to improve traffic efficiency. Wang et al. [26] constructed a two-stage SE-SBM model to measure the work performance of electronic manufacturing service providers. Because the SE-SBM model has been widely popularized in various fields, and good results have been achieved. As the research on carbon emission efficiency mainly focuses on national [27–29], interprovincial scale [30, 31], and urban scale [32–34], less attention is paid to the river basin scale. Although there has been evaluation and analysis on the Yangtze River economic belt [35–37] as an essential energy consumption base, the YRB has not made targeted research results.

Therefore, based on the consideration of carbon emission constraints and regional heterogeneity, this paper first combines the standard frontier model with the SE-SBM model considering unexpected output, calculates the carbon emission efficiency of YRB from static and dynamic perspectives, and obtains the temporal and spatial evolution characteristics of carbon emission efficiency of different provinces in the YRB. Compare various provinces’ Malmquist index decomposition results and put forward targeted suggestions. Secondly, the Kernel density function deeply analyzes the dynamic evolution characteristics of YRB’s carbon emission efficiency. Finally, combined with the Tobit model, the driving factors affecting YRB and China’s carbon emission efficiency are compared and analyzed. The research results have a particular guiding significance for improving the carbon emission efficiency of YRB.

## 3. Data processing and methodology

### 3.1. Data processing and data sources

#### 3.1.1. Measurement of the carbon emission

The carbon dioxide emissions are mainly based on the calculation method provided by the Intergovernmental Panel on Climate Change(IPCC)[38]. The calculation formula is as follows:

$$C_{j}=\sum_{i=1}^{8}E_{ij}*K_{i1}*K_{i2}$$ (1)

Where C_j_ represent the carbon emissions of eight fossil fuels in *j* province, and *E*_*ij*_ represents the consumption of the *i*th fossil fuel in province *j*. K_i1_ indicates the standard coal conversion coefficient of fossil fuel i, and K_i2_ denotes the carbon emission coefficient of fossil fuel i. The correlation coefficient of relevant fossil fuels is shown in Table 1.

**Table 1:** The correlation coefficient of relevant fossil fuels.

Coefficient type	Coal	Coke	Crude oil	Gasoline	Kerosene	Diesel oil	Fuel oil	Natural gas
K_1_	0.7559	0.8550	0.5857	0.5538	0.5714	0.5921	0.6185	0.4483
K_2_	0.7143	0.9714	1.4286	1.4174	1.4174	1.4571	1.4286	1.3300

Data sources: China Energy Statistical Yearbook.

#### 3.1.2. Selection of relevant indicators

Combined with previous relevant studies on carbon emission efficiency calculation [39–41], the input-output indicators selected in this paper are shown in Table 2. Among them, the labor force is expressed by the number of urban employees; energy consumption refers to the total consumption of the above eight main energy consumption varieties; GDP is the actual GDP based on 2005, and carbon emission is the carbon emission generated by the eight primary energy consumption. The calculation formula of capital stock is as follows:

$${CS}_{it}=(1-\delta_{i}){CS}_{i,t-1}+{IFA}_{it}$$ (2)

$${CS}_{i0}=\frac{{IFA}_{i1}}{\rho_{i}+\delta_{i}}$$ (3)

Where CS_it_ and CS_i,t−1_ respectively represent the capital stock in *t* and *t*−1 periods, and CS_i0_ indicates the initial capital stock. IFA_it_ denotes the total investment in fixed assets after adjustment in period *t*. δ_i_ and ρ_i_ respectively represent the asset depreciation rate and the average annual investment growth rate under a fixed price, in which the value of δ_i_ in this paper is 9.6%.

**Table 2:** The statistical description of the input-output indicators from 2005–2017.

Factors	Indexes	Unit	Mean	Standard deviation	Minimum	Maximum
Input factors	Capital stock	100 million yuan	7935.18	7184.31	857.00	27707.00
	Labor	10 thousand people	450.32	348.24	42.60	1290.60
	Energy consumption	million tons	33849.93	24473.94	1669.12	94550.07
Expected output	Economic output	100 million yuan	12256.23	12805.95	543.32	61470.90
Unexpected output	Carbon emissions	million tons	26503.59	19222.67	1204.47	71072.92

#### 3.1.3. Data sources

The data selected in this paper are mainly shown in Tables 1 and 2, and Section 3.2.4. The data are from China Statistical Yearbook (2006–2018), China Energy Statistical Yearbook (2006–2018), and IPCC.

### 3.2. Methodology

#### 3.2.1. Construction of Tapio decoupling model for nine provinces of YRB

In recent years, some scholars at home and abroad have used the concept of "decoupling" and related indicators to reflect the relationship between carbon dioxide and economic growth [42,43]. The Tapio model is classified according to the decoupling elasticity index. The decoupling states are subdivided into eight states: strong decoupling, weak decoupling, weak negative decoupling, strong negative decoupling, expansionary negative decoupling, expansionary connection, recessive decoupling, and recessive connection. Since the carbon emission and GDP growth of YRB are positive during the study period, these four states (strong negative decoupling, weak negative decoupling, recessive connection, and recessive decoupling) are not available in this article. To reflect the decoupling state between YRB’s carbon dioxide emission and GDP, this paper calculates the elasticity of the decoupling state between total carbon dioxide emission and GDP. The calculation formula of the elastic value of the Tapio decoupling model is as follows:

$$\delta_{i}=\frac{\Delta {CO}_{2_{i}}\%}{\Delta {GDP}_{i}\%}$$ (4)

Where, *δ*_*ij*_ represents the decoupling elasticity value of *i* province, $\Delta {CO}_{2_{i}}\%$ and Δ*GDP*_*i*_% respectively represents the growth of total carbon emissions and GDP from the base period to the end of the period of *i* province. Strong decoupling (SD) refers to the continuous positive growth of GDP and negative growth of carbon emissions, the value range of *δ*_*ij*_ is (- ∞, 0); Weak decoupling (WD) refers to the situation that both GDP and carbon dioxide emissions show growth, but the growth rate of carbon emissions is less than that of GDP, the value range of *δ*_*ij*_ is (0,0.8); Expansionary connection (EC) means that both carbon emissions and GDP show positive growth, and their growth rates are roughly the same, the value range of *δ*_*ij*_ is (0.8,1.2); Expansionary negative decoupling (end) refers to the phenomenon that carbon emissions grow faster than GDP, the value range of *δ*_*ij*_ is (1.2, + ∞) [44].

#### 3.2.2 Change of carbon emission efficiency of YRB

*3*.*2*.*2*.*1 Construction of the SE-SBM model based on the unexpected output*. As an essential tool of efficiency research, the DEA model was initially proposed by operational research experts. Because it can determine the frontier of non-parametric effective production and evaluate the relative effectiveness of decision-making units with multiple input-output indicators. It has been widely applied in carbon emissions, land use, industry management, etc. To analyze the temporal and spatial characteristics of regional carbon emission efficiency and regional carbon emission performance differences, this paper mainly introduces three models for analysis: (1) The SE-SBM model based on unexpected output is presented, which not only considers the expected output but also fully considers the impact of unexpected output. This model is different from the traditional DEA model, which allows the efficiency value of the decision-making unit to be greater than or equal to 1, which can more clearly distinguish the differences in carbon emission efficiency in different regions. The model construction of this paper mainly refers to Tone and Wang et al. [45, 46]. The model construction steps are as follows. Assuming that there are *n* decision-making units, the input of each decision-making unit is *m*, the expected output and the unexpected output are *q*_1_ and *q*_2_ respectively, which are expressed as *x*∈*R*^*m*^, $y^{d}\in R^{q_{1}},\;y^{u}\in R^{q_{2}}$. Where, *X*^*m*^, *Y*^*d*^, and *Y*^*u*^ represent the matrix of input index, expected output, and unexpected output, respectively. The three matrix expressions:

$$X=[x_{1},\cdots ,x_{n}]\in R^{m\times n},\;Y^{d}=[y_{1}^{d},\cdots ,y_{n}^{d}]\in R^{q_{1}\times n},\;Y^{u}=[y_{1}^{u},\cdots ,y_{n}^{u}]\in R^{q_{2}\times n}$$ (5)

Since it is adequate to define decision-making unit when discussing the super-efficiency SBM, so the SE- SBM model constructed in this paper can be expressed as follows:

$$\rho^{*}=\min\frac{\frac{1}{m}\sum_{i=1}^{m}\frac{\bar{x}}{x_{ik}}}{\frac{1}{q_{1}+q_{2}}(\sum_{s=1}^{q_{1}}\frac{{\bar{y}}^{d}}{y_{\mathrm{SK}}^{d}}+\sum_{t=1}^{q_{2}}\frac{{\bar{y}}^{u}}{y_{\mathrm{tk}}^{u}})}$$

$$s.t.\left\{ \begin{array}{l} \bar{x}\geq \sum_{j=1,j\neq k}^{n}\phi_{j}x_{ij},\;i=1,\;2,\;\cdots ,\;m\\ {\bar{y}}^{d}\leq \sum_{j=1,j\neq k}^{n}\phi_{j}y_{sj}^{d},\;s=1,\;2,\;\cdots ,\;q_{1}\\ {\bar{y}}^{u}\geq \sum_{j=1,j\neq k}^{n}\phi_{j}y_{tj}^{u},\;t=1,\;2,\;\cdots ,\;q_{2}\\ \bar{x}\geq x_{k},{\bar{y}}^{d}\leq y_{k}^{d},{\bar{y}}^{u}\geq y_{k}^{u},\phi_{j}\geq 0 \end{array}\right.$$ (6)

Where the ρ* denotes the efficiency value of the decision-making unit, that is, the calculated value of the SE-SBM model. The value is greater than zero. Unlike the traditional DEA model, the value can also be greater than 1, which can more clearly distinguish the difference in carbon emission efficiency between regions.

*3*.*2*.*2*.*2 Calculation of Malmquist index based on SE-SEM*. In this paper, the Malmquist index is also used to analyze the change rate of carbon emission efficiency in the YRB. By introducing the Malmquist index, the time series dynamic analysis of regional carbon emission efficiency is carried out, mainly adopting the Malmquist index model constructed by Zhou et al. [47]. Based on previous studies [48], this paper calculates the Malmquist index based on the SE-SEM model. The specific formula is as follows:

$$M(x^{t},\;y^{t},x^{t+1},\;y^{t+1})=\{\frac{\vec{D_{c}^{t}}(x^{t+1},y^{t+1})}{\vec{D_{c}^{t}}(x^{t},y^{t})}{*\frac{\vec{D_{c}^{t+1}}(x^{t+1},y^{t+1})}{\vec{D_{c}^{t+1}}(x^{t},y^{t})}\}}^{\frac{1}{2}}$$ (7)

When the M(*x*^t^,*y*^t^,*x*^t+1^,*y*^t+1^)>1, it indicates that total factor productivity level increases from *t* to *t*+1, otherwise when M(*x*^t^,*y*^t^,*x*^t+1^,*y*^t+1^)<1, it indicates that productivity level decreases. The specific Malmquist index decomposition results are as follows:

$$M(x^{t},\;y^{t},x^{t+1},\;y^{t+1})=TPC*TEC=\{\frac{\vec{D_{c}^{t+1}}(x^{t+1},y^{t+1})}{\vec{D_{c}^{t}}(x^{t},y^{t})}*[\frac{\vec{D_{c}^{t}}(x^{t+1},y^{t+1})}{\vec{D_{c}^{t+1}}(x^{t+1},y^{t+1})}*{\frac{\vec{D_{c}^{t}}(x^{t+1},y^{t+1})}{\vec{D_{c}^{t+1}}(x^{t},y^{t})}\}}^{\frac{1}{2}}$$ (8)

Where *TPC* represents the change index of technological progress. If TPC> 1, it means that the closer the decision-making unit is to the production frontier, the technological progress will be improved. Conversely, when *TPC*<1, the decision-making unit is not ideal for the existing technological innovation. *TEC* is the change index of technical efficiency. When *TEC*>1, the technical efficiency has been improved. On the contrary, the technical efficiency needs to be improved.

#### 3.2.3. Kernel density estimation method

To get the sample set’s distribution density function for a given sample set, we usually use the following two ways: (1) Parameter estimation method. It mainly includes likelihood estimation, Gaussian mixture estimation, and so on. The disadvantage of the parameter estimation method is that it needs to add subjective factors in advance, so it isn’t easy to fit the model similar to the accurate distribution. (2) Non-parametric estimation. Unlike parameter estimation, it does not need to add any prior knowledge. It only needs to fit the distribution according to the data’s characteristics and properties, getting a better model than the parameter estimation method. Among them, kernel density estimation is widely used. Kernel density estimation is a non-parametric test method for estimating the probability density function. Its advantage is that it can analyze the distribution of events according to the data’s characteristics and has no requirement for the function form [49]. This method has been widely used in academic circles, such as using the kernel density estimation method to analyze the hot spots of road traffic accidents [50], the impact of renewable energy growth on the adequacy of power generation systems [51], and the travel characteristics of Chengdu residents [52]. Based on previous studies, this paper uses the kernel density estimation method to fit the carbon emission efficiency of the YRB, obtains its probability distribution curve, and then analyzes the evolution trajectory of carbon emission efficiency in this region. The specific calculation method is as follows:

$$f(p)=\frac{1}{\mathrm{nh}}\sum_{i=1}^{n}g(\frac{p‐p_{i}}{h})$$ (9)

Where f(p) indicates the kernel density estimate, p denotes the variable, p_i_ means the marker point, n represents the number of all samples, g represents the kernel function, and *h* indicates the bandwidth. The selection of h will affect the smoothness of distribution density estimation. This paper uses the default width and process of the Eviews software.

#### 3.2.4. An empirical model of carbon emission efficiency drivers of YRB

Referring to the kinds of literature, it can be concluded that scholars use different methods to analyze the influencing factors of efficiency value in various fields [53–58]. Through the calculation of Section 3.2.1–3.2.3, this paper obtains the carbon emission efficiency value and dynamic evolution characteristics of YRB from 2005 to 2017. To further analyze the influencing factors of carbon emission efficiency, based on the perspective of regional heterogeneity, this paper selects the resident population at the end of the year to represent the regional population scale (P_1_); Regional GDP represents the degree of economic development (P_2_); The proportion of industrial GDP represents the level of industrialization (P_3_); The proportion of tertiary industry reflects the industrial structure (P_4_); The ratio of thermal power generation to total power generation represents the level of green development (P_5_); The amount of invention patent authorization represents the regional innovation ability (P_6_); The ratio of coal consumption to total energy consumption represents the level of energy consumption (P_7_), and the proportion of the urban population to total population represents the level of urbanization (P_8_); The total environmental protection expenditure reflects the regional environmental protection awareness (P_9_).

Considering that the carbon emission efficiency calculated by SE-SBM is greater than 0 and has the truncation property if the least square method is used, the model will be wrongly set, and a consistent estimator cannot be obtained. Therefore, based on the concept of the maximum likelihood method, the Tobit model is introduced in this paper. The model formula is as follows:

$${CE}_{jt}=\alpha_{0}+\alpha_{1}P_{1jt}+\alpha_{2}P_{2jt}+\cdots +\alpha_{i}P_{ijt}+\varepsilon_{jt}$$ (10)

Where *α*_*i*_ represents the parameter to be estimated, *ε*_*jt*_ represents the random error term and follows the normal distribution. To eliminate the heteroscedasticity of the data and not change the nature and relationship of the data, the natural logarithm of the dependent variable data is taken.Therefore, the regression model in this paper is modified as follows:

$${CE}_{jt}=\alpha_{0}+\alpha_{1}{lnP}_{1jt}+\alpha_{2}lnP_{2jt}+\cdots +\alpha_{i}lnP_{ijt}+\varepsilon_{jt}$$ (11)

The meaning represented by letters is the same as the above.

## 4. Results and discussion

### 4.1. Analysis of decoupling results

Due to space constraints, this paper divides the decoupling status analysis into four stages: 2005–2010, 2010–2015, 2015–2017, and 2005–2017 (Table 3).

**Table 3:** Decoupling state for YRB regions over 2005–2017.

Year	Index change	Shanxi	Inner Mongolia	Shandong	Henan	Sichuan	Shaanxi	Gansu	Qinghai	Ningxia
2005–2010	∆GDP (%)	57.49	114.7	67.1	83.36	90.11	88.15	70.61	77.3	73.72
	△CO_2_ (%)	26.26	60.16	32.88	39.83	86.17	56.02	17.84	64.33	40.05
	Elastic decoupling	0.46	0.52	0.49	0.48	0.96	0.64	0.25	0.83	0.54
	Decoupled state	WD	WD	WD	WD	EC	WD	WD	EC	WD
2010–2015	∆GDP (%)	41.47	58.7	55.39	58.67	65.42	65.52	66.13	63.77	58.64
	△CO_2_ (%)	5.39	10.95	-7.12	-3.51	18.17	23.6	25.35	57.87	49.62
	Elastic decoupling	0.13	0.19	-0.13	-0.06	0.28	0.36	0.38	0.91	0.85
	Decoupled state	WD	WD	SD	SD	WD	WD	WD	EC	EC
2015–2017	∆ GDP (%)	25.75	23.15	28.99	34.04	35.17	32.79	25.49	31.32	31.08
	△CO_2_ (%)	-2.24	22.58	-9.9	-21.4	-16.06	-10.82	-12.82	4.64	37.94
	Elastic decoupling	-0.09	0.98	-0.34	-0.63	-0.46	-0.33	-0.5	0.15	1.22
	Decoupled state	SD	EC	SD	SD	SD	SD	SD	WD	END
2005–2017	∆GDP (%)	180.17	319.64	234.95	289.96	325.08	313.53	255.66	281.3	261.24
	△CO_2_ (%)	30.08	117.83	11.2	6.05	84.66	71.97	28.77	171.46	189.04
	Elastic decoupling	0.17	0.37	0.05	0.02	0.26	0.23	0.11	0.61	0.72
	Decoupled state	WD	WD	WD	WD	WD	WD	WD	WD	WD

It can be seen that the relationship between carbon dioxide emissions and GDP in most provinces from 2005 to 2010 is in a weak decoupling state, and only Sichuan and Qinghai provinces are in an expansionary connection state, which shows that the growth of carbon emissions in most provinces during this stage is slower than that of GDP. Gansu’s decoupling elastic value is only 0.25, which indicates that the province’s carbon dioxide emissions are relatively low while achieving the same economic growth. Half of the provinces in 2010–2015 are still in a weak decoupling state, while Shandong and Henan are in a strong decoupling state, indicating that the economic growth pattern of the two provinces is resource-saving and environment-friendly. Ningxia has changed from weak decoupling to expansionary linkage due to the significant slowdown of economic growth and the slight increase of carbon emissions during this period. From 2015 to 2017, more than half of the regions showed strong decoupling. Ningxia’s GDP growth rate decreased significantly during this period, resulting in an expansionary negative decoupling state. The growth rate of carbon emission in the province was greater than that of GDP. Due to the sharp decline of GDP in Inner Mongolia, the decoupling elasticity index became 0.98

Throughout the study period from 2005 to 2017, nine provinces of YRB showed weak decoupling. Overall, the dependence of GDP growth on fossil energy in the nine provinces of YRB is declining. The economic growth pattern is developing from extensive to intensive, and the overall economic growth mode is extending to resource-friendly.

### 4.2 Results of carbon emission efficiency

This paper considers the unexpected output in the SE-SBM model based on the traditional DEA model. It uses MAXDEAPro5.0 software to calculate the carbon emission efficiency of the YRB. The results are shown in Table 4.

**Table 4:** The carbon emission efficiency of the YRB in 2005–2017.

Year	Shanxi	Inner Mongolia	Shandong	Henan	Sichuan	Shaanxi	Gansu	Qinghai	Ningxia
2005	1.00	0.61	0.61	0.63	0.63	0.56	0.58	1.10	1.00
2006	1.00	0.61	0.65	0.66	0.65	0.59	0.61	1.00	0.96
2007	1.00	0.64	0.68	0.70	0.68	0.63	0.65	1.00	1.00
2008	0.90	0.69	0.72	0.74	0.70	0.67	0.68	0.98	0.93
2009	0.84	0.74	0.75	0.77	0.74	0.71	0.70	0.97	0.94
2010	0.87	0.79	0.79	0.81	0.78	0.75	0.74	0.98	0.92
2011	0.94	0.85	0.82	0.83	0.82	0.78	0.77	0.92	1.01
2012	0.94	1.01	0.87	0.85	0.87	0.81	0.80	0.94	0.90
2013	0.97	0.87	0.89	0.84	0.84	0.82	0.80	0.95	0.91
2014	1.00	0.91	1.00	0.88	0.88	0.85	0.82	0.97	0.94
2015	0.93	1.00	1.00	0.91	0.92	0.90	0.85	0.98	0.94
2016	0.91	1.00	1.02	0.95	0.96	0.95	0.89	1.00	0.95
2017	1.08	1.06	1.05	1.03	1.06	1.01	0.90	1.03	1.12
Mean	0.95	0.83	0.83	0.82	0.81	0.77	0.75	0.99	0.96

The data in Table 4 can directly reflect the distribution of carbon emission efficiency and the evolution law of time series in the YRB from 2005 to2017. Overall, the average carbon emission efficiency of different regions in the YRB varies significantly during the study period. Shanxi, Qinghai, and Ningxia have higher carbon emission efficiency values as high as 0.95, 0.99, and 0.96. However, Shaanxi and Gansu’s carbon emission efficiency is 0.77 and 0.75, far lower than other regions. There is great potential to improve the efficiency of carbon emission reduction. In terms of time series, the carbon emission efficiency of most areas has been significantly improved. Inner Mongolia, Shandong, Henan, and Sichuan’s carbon emission efficiency had increased from about 60% in 2005 to about 103% in 2017. During the study period, the carbon emission efficiency of Gansu has also increased by 32%. However, the region’s carbon emission efficiency is still less than 1 in 2017, and there is still tremendous pressure to reduce emissions.

**Fig 3:**
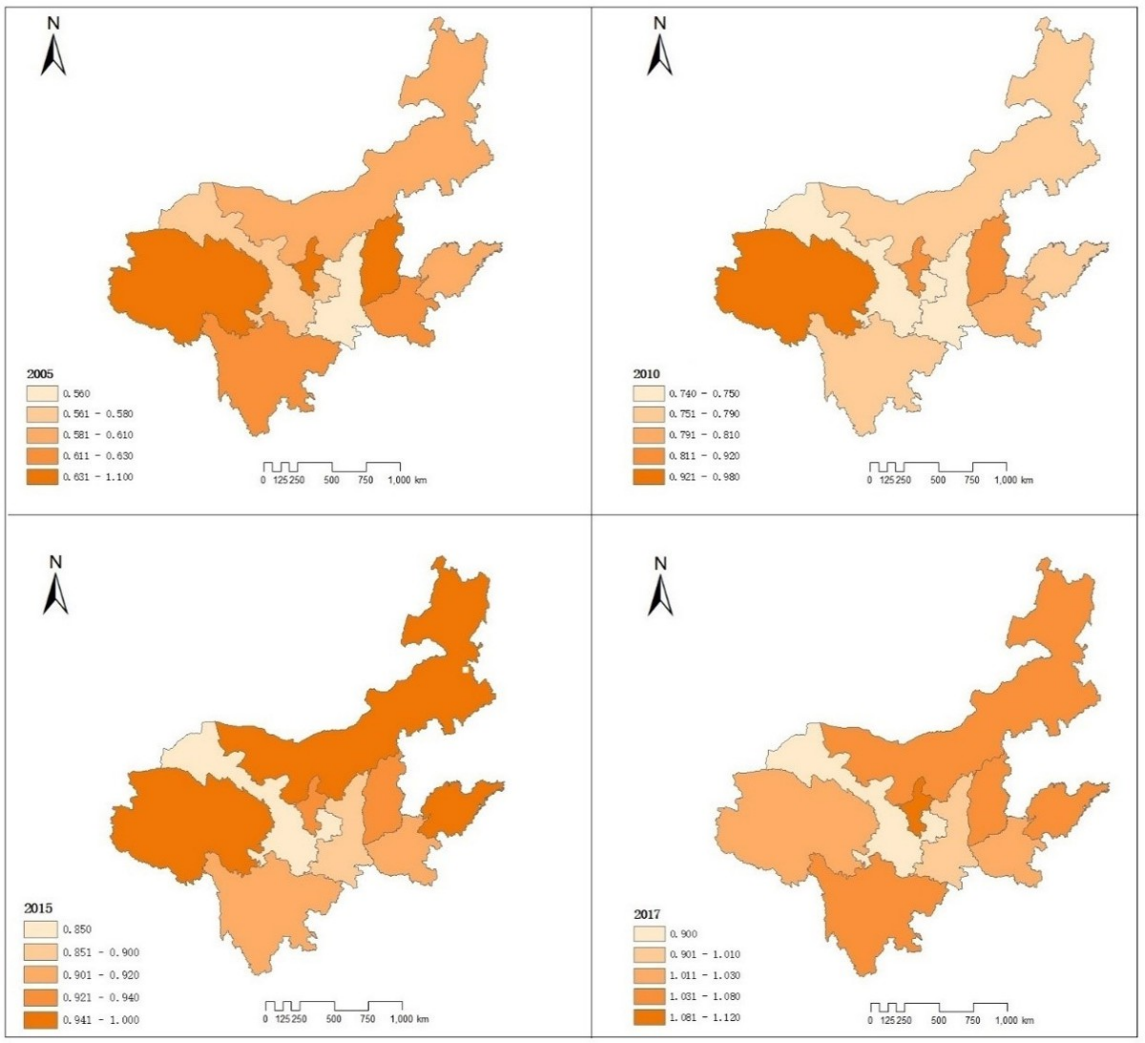
Spatiotemporal change of carbon emission efficiency in the YRB.

To more intuitively reflect the spatial heterogeneity of carbon emission efficiency in the YRB, the region’s carbon emission efficiency charts in 2005, 2010, 2015, and 2017 were drawn (Fig 3). As can be seen from the figure, the regions with the highest carbon emission efficiency in 2005 were Shanxi, Qinghai, and Ningxia. Their carbon emission efficiency was significantly higher than 1. Gansu and Shaanxi have the lowest carbon emission efficiency score, no more than 0.6. The regions with high carbon emission efficiency in 2010 were similar to those in 2005. Shanxi, Qinghai, and Ningxia still had the highest carbon emission performance, but the carbon emission efficiency value dropped to 0.87,0.98, 0.98, respectively.

In comparison, the carbon emission efficiency values of the other six provinces have increased to varying degrees. In 2015, the provinces with the highest carbon emission efficiency were Inner Mongolia and Shandong, which reached 1. And the carbon emission efficiency value of other regions is basically above 0.9, which indicates that the carbon emission performance of the YRB region has been significantly improved, which is closely related to the unification of the national carbon market in 2013. In 2017, only Gansu province’s carbon emission efficiency value was lower than 1, among which the carbon emission efficiency value of Ningxia was as high as 1.12. until 2017, the carbon emission efficiency value of most of the YRB was more significant than 1, which indicates that under the background of the national carbon peak in 2030 and carbon neutral in 2060, the region has continuously improved the carbon emission efficiency and made a particular contribution to the national carbon emission reduction target.

#### 4.2.1 Static analysis results of carbon emission efficiency

**Fig 4:**
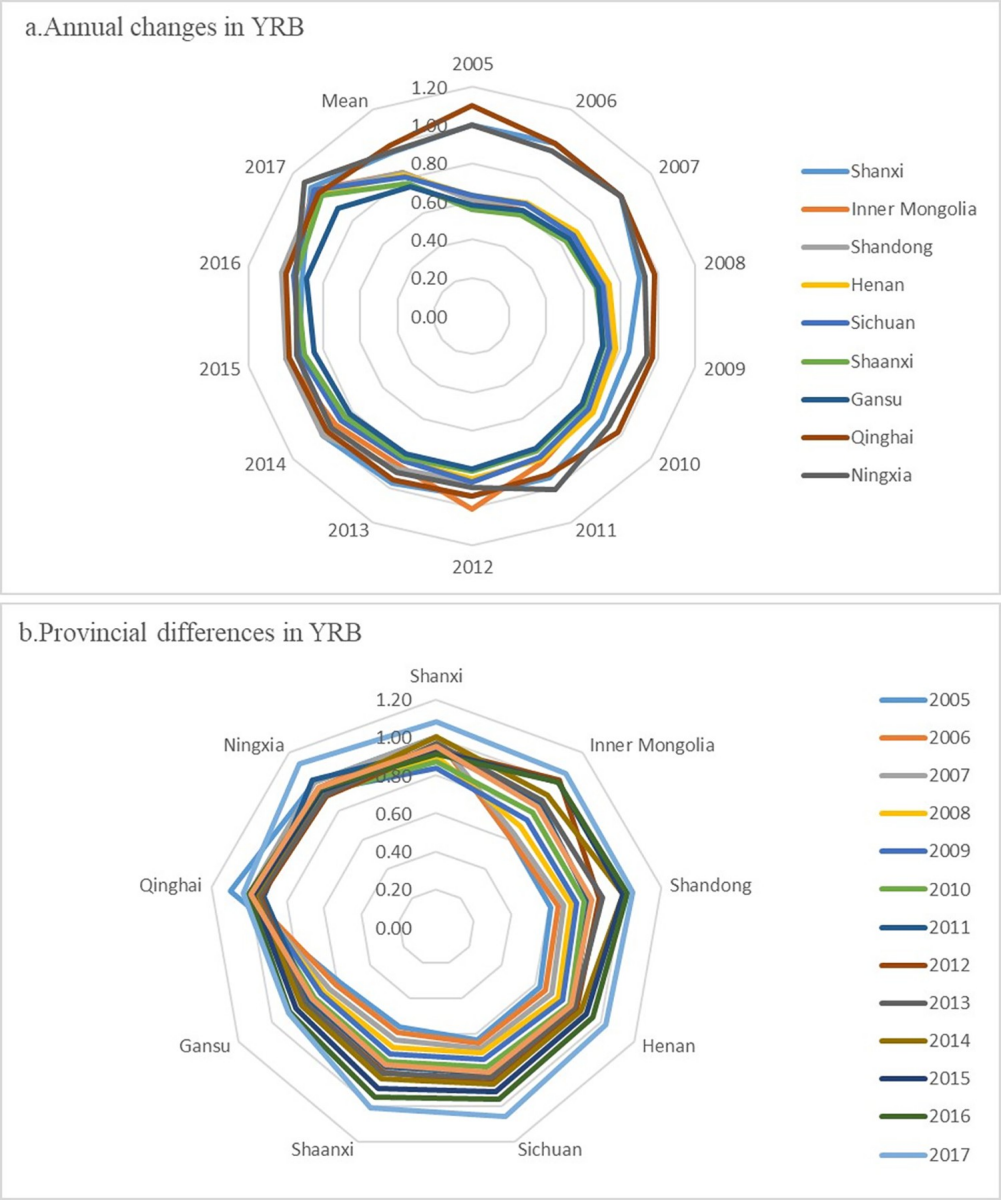
Radar chart of comprehensive energy efficiency in the YRB.

As shown in Fig 4A, each province’s carbon emission efficiency has been improved to a certain extent. With the national carbon market promotion in 2013, China entered a normal development stage, and the economy gradually shifted from high-speed growth to a high-quality development stage. Besides, due to our government’s attention to the ecological protection and high-quality development of the YRB, this region’s carbon emission efficiency has been significantly improved.

From 2005 to 2017, the carbon emission efficiency of the YRB varies significantly among provinces, and the annual variation range of carbon emission efficiency of most regions is significant. There are apparent differences in regional carbon emission efficiency, as shown in Fig 4B. Inner Mongolia, Shandong, Sichuan, and Shaanxi have considerable carbon emission efficiency. Shanxi, Qinghai, and Ningxia’s carbon emission efficiency shows a fluctuating slow-rising trend of "high-low-high."

**Fig 5:**
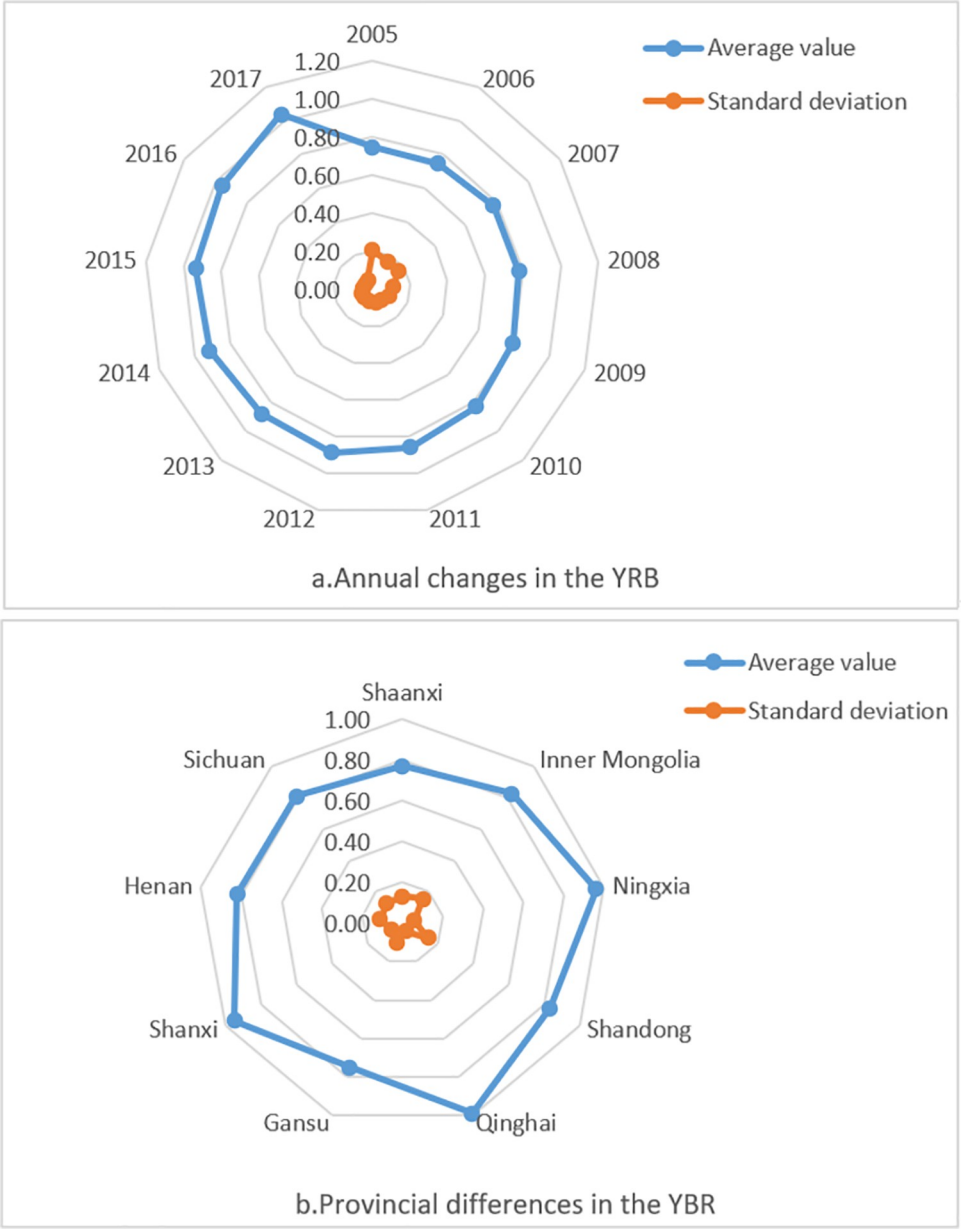
Changes in carbon emission efficiency in the YRB during 2005–2017.

To more clearly analyze the differences of carbon emission efficiency on the time scale and spatial scale in the YRB, Fig 5 is drawn, which are annual carbon emission efficiency change (Fig 5A) and carbon emission efficiency change of different provinces (Fig 5B). As shown in Fig 5A, each year’s average carbon emission efficiency from 2005 to 2017 increased from 0.747 to 1.038, indicating a slow upward trend. Over time, the standard deviation of carbon emission efficiency decreased from 0.206 to 0.057. The dispersion degree continued to decline, meaning that each province’s carbon emission efficiency difference decreased with time. Fig 5B shows that each region’s average carbon emission efficiency differs. Ningxia, Shanxi, and Qinghai’s average carbon emission efficiency is higher than 0.95, and the standard deviations of carbon emission efficiency of these three provinces are 0.056, 0.043, and 0.062, respectively. These provinces’ dispersion degree is shallow, which indicates that these provinces’ carbon emission efficiency is rising slowly. On the contrary, Shanxi’s standard deviation of carbon emission efficiency is as high as 0.132. The average value is only 0.77, which indicates that Shanxi’s carbon emission efficiency varies significantly from year to year.

#### 4.2.2. Dynamic analysis results of carbon emission efficiency

Based on the analysis of the static characteristics of carbon emission efficiency in the YRB by the SE-SBM model considering the unexpected output in Section 3.2, this paper further analyzes the dynamic change characteristics of carbon emission efficiency in the YRB by using the Malmquist index, as shown in Table 5.

**Table 5:** Results and decomposition of Malmquist index.

Regions	Rate of change index	2005–2006	2006–2007	2007–2008	2008–2009	2009–2010	2010–2011	2011–2012	2012–2013	2013–2014	2014–2015	2015–2016	2016–2017	Mean
Shanxi	M	1.00	1.00	0.90	0.94	1.03	1.08	1.00	1.02	1.03	0.93	0.98	1.19	1.02
	TEC	0.97	0.96	0.92	0.95	0.98	0.94	0.99	1.05	0.99	0.99	0.98	1.06	0.98
	TPC	1.03	1.05	0.98	0.98	1.06	1.15	1.01	0.98	1.04	0.93	1.00	1.12	1.04
Inner- Mongolia	M	0.99	1.05	1.07	1.08	1.07	1.07	1.19	0.86	1.04	1.10	1.00	1.06	1.05
	TEC	1.02	1.02	1.05	1.05	1.02	1.05	1.03	0.95	1.00	1.00	1.01	0.98	1.01
	TPC	0.97	1.03	1.02	1.03	1.05	1.02	1.16	0.91	1.04	1.10	0.99	1.08	1.03
Shandong	M	1.06	1.06	1.05	1.05	1.04	1.04	1.06	1.03	1.12	1.00	1.02	1.03	1.04
	TEC	1.01	0.99	1.03	1.00	1.01	0.97	1.08	0.98	1.05	1.03	1.02	0.99	1.02
	TPC	1.05	1.07	1.02	1.05	1.03	1.07	0.99	1.05	1.07	0.97	0.99	1.03	1.03
Henan	M	1.05	1.06	1.05	1.05	1.05	1.02	1.03	0.99	1.04	1.04	1.04	1.08	1.05
	TEC	1.00	1.00	1.00	1.00	1.00	1.00	0.99	1.00	1.00	1.00	1.00	1.01	1.00
	TPC	1.05	1.06	1.05	1.05	1.05	1.02	1.04	0.99	1.04	1.04	1.04	1.08	1.05
Sichuan	M	1.04	1.05	1.03	1.05	1.05	1.06	1.05	0.97	1.05	1.04	1.05	1.10	1.05
	TEC	1.01	0.99	0.99	0.97	1.04	1.04	0.99	1.01	0.98	1.03	1.01	1.02	1.01
	TPC	1.03	1.05	1.05	1.08	1.01	1.02	1.07	0.97	1.06	1.01	1.04	1.09	1.04
Shaanxi	M	1.05	1.07	1.07	1.06	1.06	1.04	1.04	1.01	1.04	1.05	1.06	1.07	1.05
	TEC	0.99	1.00	1.02	1.02	1.02	1.01	1.03	1.02	1.01	1.00	1.01	1.00	1.01
	TPC	1.05	1.07	1.05	1.04	1.03	1.03	1.02	0.99	1.04	1.04	1.05	1.07	1.04
Gansu	M	1.05	1.06	1.05	1.04	1.05	1.04	1.04	1.00	1.03	1.04	1.04	1.01	1.03
	TEC	1.00	1.00	1.00	1.00	0.92	1.01	1.07	1.00	1.00	1.00	1.00	0.89	0.98
	TPC	1.06	1.06	1.05	1.04	1.14	1.03	0.97	1.00	1.03	1.03	1.04	1.13	1.05
Qinghai	M	0.92	1.00	0.98	0.98	1.01	0.94	1.02	1.01	1.02	1.02	1.02	1.03	1.01
	TEC	0.92	1.01	0.91	1.04	1.11	1.05	0.93	0.98	1.09	1.09	0.86	1.20	1.02
	TPC	1.00	0.99	1.09	0.94	0.91	0.90	1.09	1.02	0.94	0.93	1.18	0.86	0.99
Ningxia	M	0.96	1.04	0.93	1.02	0.97	1.10	0.89	1.01	1.04	1.00	1.01	1.17	1.02
	TEC	0.99	1.06	1.02	1.07	1.07	1.06	0.95	1.02	1.01	1.02	0.98	1.06	1.03
	TPC	0.97	0.98	0.91	0.95	0.91	1.03	0.93	0.98	1.02	0.98	1.04	1.10	0.99

The Malmquist index showed a fluctuating downward trend only in Shandong and Gansu from the time dimension analysis. The Malmquist index’s decomposition value indicates that the decrease in their overall carbon emission efficiency is mainly due to the decline of the TEC. The other seven regions’ carbon emission efficiency showed a fluctuating upward trend, of which the most significant increase was in Shanxi. Its Malmquist index rose from 1.00 in 2005 to 1.197 in 2017, mainly due to the sharp rise of TPC, which indicated that the rapid technological progress in the region promoted the improvement of carbon emission efficiency.

From the average point of view, Qinghai and Ningxia are different from other regions. The decline of the TPC in these two regions hinders the improvement of the Malmquist index. It shows that these two regions should pay more attention to technological progress in future development, accelerate low-carbon technology growth, and encourage cleaner production. The decrease of technical efficiency index TEC in Shanxi and Gansu is the main reason to hinder carbon emission efficiency. This shows that the two regions should improve the technical efficiency by enhancing the pure technical efficiency or expanding the scale of technology used to promote overall efficiency.

### 4.3 Dynamic evolution characteristics of carbon emission efficiency in YRB

**Fig 6:**
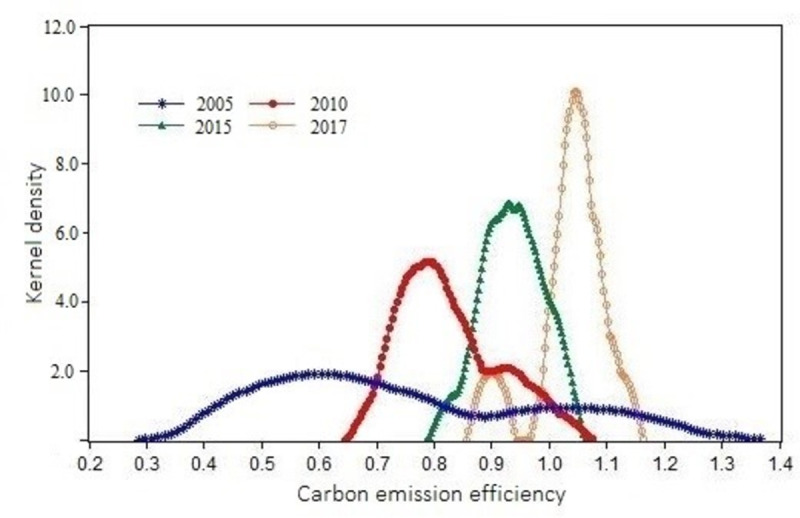
Dynamic evolution characteristics of carbon emission efficiency in YRB.

This paper selects the dynamic evolution characteristics of carbon emission efficiency values of 2005, 2010, 2015, and 2017 (Fig 6) and analyzes the corresponding years’ kernel density distribution. The results are as follows:

From the kernel density curve’s position, the carbon emission efficiency in the YRB from 2005 to 2017 shifted to the right, and the carbon emission efficiency increased significantly. Over time, the number of high-value areas corresponding to the carbon emission efficiency kernel density curve increases, while the number of the low-value regions decreases. This changing trend fully shows that the overall carbon emission efficiency of the YRB presents a stable growth trend.

In terms of the shape of the kernel density curve, the carbon emission efficiency in the YRB from 2005 to 2017 is not strictly a single peak or multi-peak state. Still, there is a tailing phenomenon, skewed the overall distribution. This shows that there are still unreasonable development scenarios in some provinces in improving the carbon emission efficiency of YRB. During the study period, although the carbon emission efficiency of Shandong, Henan, Sichuan, Gansu, and Qinghai showed a steady improvement, the carbon emission efficiency of Shanxi, Ningxia, and Inner Mongolia in the YRB fluctuated significantly, with a large "rise-decline-rise" fluctuation, which is related to the fact that the region is a resource-based province. It is closely related to a large number of coal resources. In 2005, the distribution of carbon emission efficiency in different provinces of the YRB was quite different and showed a severe two-level differentiation. In 2010, the kernel density curve showed irregular partial normal distribution, the carbon emission efficiency was about 0.8, and the number of low-value areas increased slightly. In 2015 and 2017, the nuclear density showed irregular partial favorable distribution, but the two-stage differentiation improved significantly. By observing the kernel density curves in 2015 and 2017, it is found that their carbon emission efficiency still presents an irregular partial normal distribution. Even the two-level differentiation is significantly improved compared with 2005 and 2010.

In terms of kurtosis, the carbon emission efficiency of the YRB developed from a broad peak to a narrow ridge from 2005 to 2017. The kurtosis increased significantly, indicating that each region’s carbon emission efficiency gradually increased to a high-efficiency level. As shown in Fig 4, the curve area of carbon emission efficiency in 2005 is enormous. It has a broad peak, indicating that the development trend of carbon emission efficiency in most regions in this period is the same. The carbon emission efficiency curve developed from a broad peak to a narrow peak in the other three years, and the kurtosis also increased significantly. The proportion of provinces with high carbon emission efficiency corresponding to the peak also increased significantly.

### 4.4 Empirical analysis of driving factors

The regression results of the Tobit model can be obtained according to Formula 10–11, as shown in Table 6.

**Table 6:** Tobit model regression results.

Index	Regression coefficient	Standard error	z-value	p-value	95% CI
Intercept	-1.12	0.061	-18.398	0	-1.239 ~ -1.000
	-1.175*	0.033*	-35.237*	0*	-1.240 ~ -1.109*
LnP_1_	0.123	0.099	1.233	0.218	-0.072 ~ 0.318
	0.259*	0.05*	5.216*	0*	0.162 ~ 0.357*
LnP_2_	1.5	0.233	6.428	0	1.043 ~ 1.958
	0.72*	0.099*	7.281*	0*	0.526 ~ 0.914*
LnP_3_	1.21	0.303	3.994	0	0.616 ~ 1.804
	0.087*	0.094*	0.934*	0.35*	-0.096 ~ 0.271*
LnP_4_	3.291	0.37	8.901	0	2.567 ~ 4.016
	0.578*	0.133*	4.336*	0*	0.317 ~ 0.839*
LnP_5_	-0.295	0.103	2.852	0.004	-0.092 ~ -0.497
	-0.055*	0.034*	1.581*	0.114*	-0.013 ~ -0.122*
LnP_6_	0.158	0.081	1.952	0.051	-0.001 ~ 0.317
	0.06*	0.035*	1.726*	0.084*	-0.008 ~ 0.129*
LnP_7_	-0.155	0.191	-0.814	0.416	-0.530 ~ 0.219
	-0.253*	0.051*	-4.924*	0*	-0.354 ~ -0.152*
LnP_8_	-2.624	0.516	-5.085	0	-3.635 ~ -1.613
	-0.597*	0.181*	-3.305*	0.001*	-0.952 ~ -0.243*
LnP_9_	0.247	0.079	3.134	0.002	0.092 ~ 0.401
	0.178*	0.022*	8.027*	0*	0.134 ~ 0.221*
Log (Sigma)	-1.12	0.061	-18.398	0	-1.239 ~ -1.000
	-1.175*	0.033*	-35.237*	0*	-1.240 ~ -1.109*

*Note*: the results with * are related to the influencing factors of carbon emission efficiency in China, and the rest are associated with the influencing factors of carbon emission efficiency in the YRB.

From the first three indicators of influence degree, the main factors affecting the carbon emission efficiency of YRB are industrial structure, urbanization level, and economic development level, and all show a significance of 0.01 level. When other conditions remain unchanged, the carbon emission efficiency increases by 3.291%, - 2.624%, and 1.5% for every 1% increase of the above three factors. The top three factors affecting China’s overall carbon emission efficiency are economic development, urbanization, and industrial structure, which also shows a significant level of 0.01. When other conditions remain unchanged, the carbon emission efficiency increases by 0.72%, - 0.597%, and 0.578% for every 1% increase of the above three factors. This shows that the main influencing factors affecting the carbon emission efficiency of YRB and China are the same, but the degree of influence is different.

From the perspective of population size, the p-value of population size on carbon emission efficiency of YRB area is 0.218 (P > 0.05), indicating that population size will not have an impact on the carbon emission efficiency of YRB. The regression coefficient of population size on China’s carbon emission efficiency is 0.259 and passed the significance test of 0.01, indicating that population size positively impacts China’s carbon emission efficiency to a certain extent. From the perspective of the industrialization level, the regression coefficient of the index on the carbon emission efficiency of YRB is 1.21. It passes the test of 0.01 significant level, while the p-value of the index on China’s carbon emission efficiency is 0.35, which gives the significance test of 5% level. Therefore, the industrialization level has no significant impact on China’s carbon emission efficiency. From the perspective of the green development level, on the premise that other conditions remain unchanged, the carbon emission efficiency declined by 0.295% for every 1% increase in the proportion of thermal power generation in YRB. However, the *p* value of the green development level on China’s carbon emission efficiency is 0.114, therefore, the degree of green development has no significant impact on China’s carbon emission efficiency. YRB’s coal consumption level fails to pass the significance test of 5%, so the coal consumption level will not significantly impact carbon emission efficiency. However, the coal consumption level increases by 1%, China’s carbon emission efficiency decreases by 0.253%, showing a significant level of 0.01.

As the YRB is the main energy production and consumption base in China, such as Shanxi and Inner Mongolia, which have a large number of coal resources, how to make efficient use of the energy use right in this region is of great strategic significance to reduce the energy vulnerability of this region [59]. Therefore, how to ensure the high-quality and low-carbon development of resource-based provinces is also the key to achieve China’s "30.60" double carbon goal.To sum up, there are substantial differences in the influencing factors and degree of carbon emission efficiency between YRB and China. Targeted emission reduction suggestions should be formulated according to the characteristics of YRB.

### 4.5 Robustness check

To test the robustness of our estimation, this paper replaces the proportion of thermal power generation with per capita power consumption. It then calculates the influence degree of influencing factors of carbon emission efficiency. The research results are shown in Table 7. The results show that the leading positive indicators affecting the carbon emission efficiency of the YRB are still the industrial structure and economic level, and the negative indicators are still the level of urbanization. By comparing the correlation coefficients and *P* values in Tables 6 and 7, it is proved that our estimation is reliable.

**Table 7:** The robustness test (the proportion of thermal power generation is replaced by per capita power consumption).

Index	Regression coefficient	Standard error	z-value	p-value	95% CI
Intercept	-1.303	0.061	-21.406	0.000	-1.422 ~ -1.183
LnP_1_	-0.399	0.272	-1.465	0.143	-0.932 ~ 0.135
LnP_2_	2.738	0.366	7.481	0.000	2.021 ~ 3.455
LnP_3_	0.682	0.365	1.867	0.062	-0.034 ~ 1.397
LnP_4_	3.732	0.762	4.896	0.000	2.238 ~ 5.226
LnP_5_	-1.072	0.223	-4.811	0.000	-1.509 ~ -0.635
LnP_6_	-0.150	0.092	-1.624	0.104	-0.331 ~ 0.031
LnP_7_	0.162	0.296	0.549	0.583	-0.418 ~ 0.743
LnP_8_	-4.338	0.725	-5.984	0.000	-5.758 ~ -2.917
LnP_9_	0.007	0.075	0.097	0.923	-0.139 ~ 0.154
log (Sigma)	-1.303	0.061	-21.406	0.000	-1.422 ~ -1.183

## 5. Conclusions and policy recommendations

### 5.1 Conclusions

Based on the panel data of nine provinces of YRB from 2005 to 2017, firstly, each province’s carbon emissions and decoupling status are calculated. Secondly, the temporal and spatial evolution characteristics of carbon emission efficiency of YRB are analyzed by the SE-SBM model and Malmquist index, and the dynamic evolution characteristics are deeply studied with the Kernel density function. Finally, taking the calculated carbon emission efficiency value as the unexplained variable, the Tobit model is constructed to analyze the influencing factors of YRB’s carbon emission efficiency. The conclusions are as follows:

1. From 2005 to 2017, the decoupling between carbon emissions and economic growth in most provinces changed from weak decoupling to strong decoupling. This shows that YRB’s economic growth dependence on fossil energy has gradually changed from strong to weak. Economic growth has changed from extensive to relatively intensive, and overall economic growth mode has developed to be resource-friendly.
2. There are noticeable differences in carbon emission efficiency among the nine provinces of YRB, and the overall carbon emission efficiency shows a stable growth trend. The results show that only the decline of the Malmquist index in Qinghai and Ningxia is mainly caused by reducing technological progress. In contrast, the improvement of the technical efficiency index in the other seven provinces is the main reason for the rise of the Malmquist index. The high-value area of carbon emission efficiency mainly increased, and the phenomenon of two-level differentiation gradually improved.
3. The industrial structure and economic development level have a significant positive impact on the carbon emission efficiency of YRB and China. In contrast, the urbanization level has a significant negative effect, but their impact degree is quite different. Unlike the influencing factors of China’s overall carbon emission efficiency, population size has no significant impact on carbon emissions in the YRB. The level of industrialization and green development has significant positive and negative effects on the carbon emission efficiency of YRB, respectively.

### 5.2 Policy recommendations

Based on the above research, this paper attempts to provide policy suggestions for improving YRB carbon emission efficiency from three angles.

1. To promote the realization of the "30.60" double carbon emission reduction target, the YRB region as an essential energy base of China should accelerate the reduction of carbon emissions while ensuring economic development. Efforts should be made to achieve YRB’s strong decoupling between carbon emissions and economic development in all provinces, especially Inner Mongolia and Ningxia. It is necessary to vigorously develop a low-carbon economy and realize strong decoupling between the two provinces as soon as possible.
2. We should accelerate the technical exchange among YRB’s provinces, promote new energy investment, actively promote new energy such as wind power, photovoltaic and solar energy, strive to control carbon emissions from the source, promote the coordinated development of various provinces in the region and jointly realize low-carbon development. Among the nine provinces, Qinghai and Ningxia should speed up technological progress to improve carbon emission efficiency, increase investment in low-carbon technologies, strengthen law enforcement, and force eliminating high-carbon technologies. The other seven provinces should expand the use scale of low-carbon technologies and improve their technical efficiency index through organizational process optimization to promote the improvement of carbon emission efficiency.
3. To optimize the industrial structure, we should actively develop high-tech industries, optimize the industrial structure, and explore the path of industrial structure optimization under the background of low-carbon development. Improve industrial management technology and promote the improvement of carbon emission efficiency of YRB by expanding the tertiary industry and forcing the continuous optimization of industrial structure. Strive to improve the economic development level of each province. With improved financial status, people’s awareness of environmental protection will continue to increase to enhance carbon emission efficiency. Control the urbanization process of each province of YRB, do an excellent job in the relevant supporting resettlement after the urban transfer, and strive to reduce the carbon emission caused by the acceleration of urbanization. In addition, increasing the total industrial output value and reducing the proportion of thermal power generation can improve the regional carbon emission efficiency to a certain extent.

### 5.3 Outlook

The purpose of this paper is to study the carbon emission efficiency and its dynamic evolution characteristics of the YRB, and find out the main reasons for the rise or decline of carbon emission efficiency in different provinces according to the changes of carbon emission efficiency in different provinces in the region. In order to further identify the influencing factors affecting the regional carbon emission efficiency, this paper introduces relevant indicators such as economic development, urbanization degree and industrialization degree for in-depth calculation, and identifies the main influencing factors affecting the regional carbon emission efficiency. In fact, with the promotion of the "30.60" double carbon goal, the energy consumption structure, the development of renewable technologies, the cost of wind power optoelectronics and CCUS technology will have a great impact on regional carbon emissions. Therefore, in the next step, we will deeply study the development of renewable resources in the YRB and the impact of CCUS and carbon sink on carbon emissions efficiency. And try to introduce the factors such as the installed capacity and estimated power generation of renewable resources in different provinces of the region into the next research work.

## Supporting information

S1 File (XLSX)
